# Association between residential greenspace structures and frailty in a cohort of older Chinese adults

**DOI:** 10.1038/s43856-022-00093-9

**Published:** 2022-04-20

**Authors:** Qile He, Hao-Ting Chang, Chih-da Wu, John S. Ji

**Affiliations:** 1 Institute of Medical Information, Chinese Academy of Medical Sciences and Peking Union Medical College, Beijing, China; 2 Department of Environmental and Occupational Health, National Cheng Kung University, Tainan, Taiwan; 3 Department of Geomatics, National Cheng Kung University, Tainan, Taiwan; 4 National Institute of Environmental Health Sciences, National Health Research Institutes, Miaoli, Taiwan; 5 Global Health Research Center, Duke Kunshan University, Kunshan, China; 6 Vanke School of Public Health, Tsinghua University, Beijing, China

**Keywords:** Lifestyle modification, Epidemiology

## Abstract

**Background:**

Frailty is a late-life clinical syndrome resulting from the accumulation of aging-induced decline. Greenspaces measured with normalized difference vegetation index (NDVI) are protective of frailty. However, NDVI is not as informative as structure indices in describing greenspaces’ constitution, shape, and connectivity measured by the largest patch index (LPI), shape index, and cohesion index representing larger, more complex, and more dense greenspaces through higher values. We aim to study the association between greenness structures and frailty in a cohort of Chinese older adults.

**Methods:**

We included older adults from 2008–2014 China Longitudinal Healthy Longevity Survey (CLHLS). We used greenspace indices from satellite to quantify structures (area-edge, shape, proximity) at county-level, and calculated frailty index (FI) as an outcome. We did cross-sectional analyses using linear and logistical regression, and longitudinal analyses using the generalized estimating equations (GEE).

**Results:**

Among 8776 baseline participants, mean LPI, shape, cohesion, and FI are 7.93, 8.11, 97.6, and 0.17. In cross-sectional analyses, we find negative dose-response relationships for greenspace structures and frailty, especially in females, centenarians, illiterate people, city residents, unmarried people, and individuals with increased frailty. Participants living in the highest quartile of LPI, shape, and cohesion have 32% (95%CI: 21–42%), 35% (95%CI: 24–44%), and 37% (95%CI: 26%–46%) lower odds of frailty than the lowest quartile. However, we do not find a significant association in longitudinal analyses.

**Conclusions:**

Higher levels of greenness structures (area-edge, shape, and proximity) might be related to lower frailty, while a clear longitudinal benefit cannot be identified in this analysis.

## Abstract (web summary)

He et al. study the relationship between residential greenspace structures and frailty in a cohort of older adults from China. In their cross-sectional analyses, the authors report that higher levels of greenness are associated with lower levels of frailty, particularly amongst certain subgroups including women and centenarians.

## Plain language summary

Areas dominated by vegetation (greenspaces) are associated with reduced frailty, an aging-related decline in physical function. Here, we aimed to look at the association between specific greenspace structures and frailty in a cohort of older Chinese adults. We use demographic and health data from a 6-year survey and greenspace structure data from satellites to conduct our statistical analysis. We find that a larger area, more complex shape, more concentrated greenspaces, and greater proximity might lower levels of frailty, especially in females, people aged 100 and above, illiterate people, city residents, unmarried people, and individuals with increased frailty. These findings help us to understand how the environment impacts our health and how we might mitigate frailty in older aged adults.

## Introduction

Frailty refers to a geriatric syndrome that increases a person’s vulnerability due to degenerative changes and chronic diseases, reflecting cumulative physical, psychological, and social health issues, which leads to higher risks of hospitalization, falls, depression, and mortality^1–3^. Frailty is a good predictor of health and well-being, representing an intermediate stage between robust health and the end of life. Urbanization and population aging from the developing world have been noticeable and renewed the interest in studying older adults’ well-being. China is one of the fastest aging countries and has the largest population over 80 years old globally^4^. As the degree of aging shows an increasing trend, the number of older adults in frail increases. A study in China reported that 7.0% of adults aged 60 years or older were frail^5^.

Greenness and greenspaces have been quantified in epidemiologic studies predominantly using a vegetation index (typically the normalized difference vegetation index (NDVI)) derived from satellite imagery, which measures light reflected from the earth’s surface during photosynthetic activity, from which vegetative density within a spatial area can be estimated^6^. There is evidence of an association between greenness and frailty-related factors in population health studies. A study in Hong Kong found that higher residential greenness levels could benefit frailty problems by mediating through physical activity, the number of diseases, and cognitive functions^7^. A longitudinal study with 16,238 older adults with a 12-year follow-up in China assessed greenery exposure at the neighborhood level, indicating that higher residential greenness levels are related to a lower likelihood of frailty, specifically in urban areas^8^. Besides, mechanisms by which exposures to greenspaces promote healthy aging have been extensively studied. First, more greenspaces in the residential environment could lead to fewer incidences of loneliness, more social support, and improved social cohesion in the neighborhood^9^. Second, greenspaces may be a resource for psychological restoration^10^. Exposure to greenspaces is associated with reduced stress and providing the opportunity to restore directed attention^11,12^, which may benefit cognitive aging^13^. Third, older adults living in areas with higher access to greenspaces do more physical activities, which play an important role in maintaining functioning and health^14^. Last, increased exposure to greenspaces has been associated with lower exposure to environmental stressors such as air pollution, noise, and heat, which are detrimental to health^15^.

Although previous researchers have identified the benefits of overall greenness measured with NDVI in different spatial scales^8^, the association between specific structures of greenness and frailty has not been well understood yet. This study provides new insights into the role of greenspace structures in preventing older adults’ frailty. Greenspace structure is an indispensable component of environmental systems and is essential to residents. Due to urbanization, people created artificial landscapes to replace natural greenspaces such as forests and grassland^16^. These urban greenspaces had various structural characters, which play an important role in environmental benefits. For example, Xiao et al. found the area and shape of greenspaces can affect the cooling effect of urban parks^17^. Rui et al. discovered the relationship between quantity and structure of residential greenspaces and the idealized microclimate^18^. Recently, increasing attention has been focused on exploring how structural characteristics of certain greenspaces can affect health benefits^19^. Some researchers argued that green structures can improve mental health^20–22^, and reduce risks of deaths due to cardiovascular and respiratory diseases^23,24^. Specifically, larger greenspace (larger area and edge) is associated with less depression and stress^25^; complex greenspace (shape) is correlated with higher preference and satisfaction^26^. Therefore, more research studies are worthy to be done to explore the further health benefits of greenness structures.

The purpose of this study is to go beyond using NDVI and incorporate greenspace structures measured with three characteristics: area-edge, shape, and proximity on the frailty of Chinese older adults by using the China Longitudinal Healthy Longevity Survey (CLHLS), a representative sample of older adults in China. Such investigations can give evidence of new exposure metrics for greenspace and health researchers. Our aim is also to assist provide evidence to urban planning policymakers to prolong healthy lifespan, and the provision of more robust scientific supports for urban design of greenspaces’ allocation.

In this study, we find a protective association of greenness structures and the frailty of older adults in the cross-sectional analysis. Larger areas, more complex shapes, and greater proximity are associated with a lower likelihood of frailty among Chinese older adults, especially among females, centenarians, illiterate people, city residents, unmarried people, and individuals with an increased degree of frailty. However, we do not find any significant association in the longitudinal analysis. Whilst this study does not confirm the longitudinal benefits and determinate causal relationship, it partially substantiates the negative relationship between greenness structures and frailty. This study also lays a path for further research to understand which characteristics of greenspaces have the most substantial influence on frailty.

## Methods

### Study population

The CLHLS was a national survey for investigating the determinants of healthy longevity among the older Chinese population in 22 provinces. The survey has drawn areas from a population base of 1.1 billion people, representing 85 percent of China’s total population. Investigators interviewed individuals about socioeconomic characteristics, lifestyle, physical capacity, cognitive function, and psychological status. The institutional review boards’ (IRB) approval and other information were described in the published cohort profile^27^. All analyses were based on a previous public cohort approved by the ethics committee (10.18170/DVN/UWS2LR), thus no ethical approval and patient consent are required in this study. This study used 2008–2014 data which consisted of 16,072 individuals who received baseline interviews in 2008/09, and follow-ups in 2012 and 2014. We excluded individuals who had missing demographic characters (*N* = 3073), frailty index (*N* = 4215), and NDVI (*N* = 8) at baseline year. There was no significant difference between baseline characters of excluded individuals and the overall sample. The final sample size was 8776 at baseline year.

### Greenness databases

**Fig. 1:**
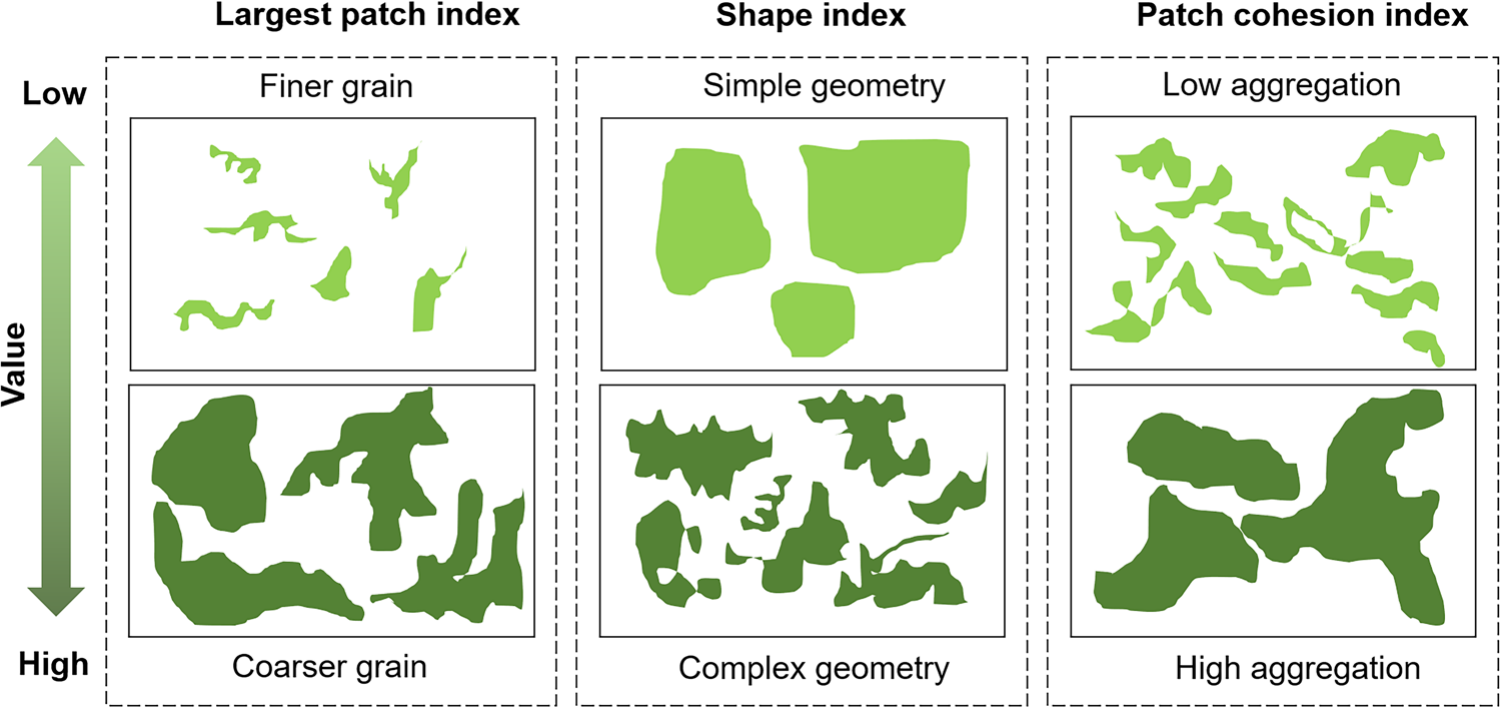
Greenspace indices.

Landscape indices were usually grouped into eight universal, and consistent characteristics to describe the major attributes of landscape structures as comprehensively as possible: area and edge, shape, contrast, core area, proximity, subdivision, isolation, and diversity^28,29^. First, considering that many indices simultaneously measured multiple aspects of structure, and some were inherently redundant because they were alternate ways of representing the same basic^29^, we used three non-redundant characters: area and edge, shape, and proximity as our exposure measurements to reflect independent and representative greenness structures, in view that these characters have been foundational in previous researches^23,30–32^. Second, McGarigal indicated that indices within the same character had sufficient similarity to represent the measurement in this category^33^, so we calculated one index (calculation unit: hectare) for each characteristic in the main model, including the largest patch index (area and edge), mean shape index (shape), and patch cohesion index (proximity) by using a software called FRAGSTATS 4.2 which was designed to compute landscape metrics for categorical map patterns (Fig. 1) (Supplementary Table 1)^34,35^. Third, Spearman’s correlation analysis was used to avoid collinearity of the three calculated indices, with the inclusion criterion for the correlation coefficient of independent variables <0.7^21^. The largest patch index (LPI, ranges from 0.01 to 53.7 in this study, mean = 8.6) shows the percentage of the landscape comprised of the largest patch. The shape index (shape, ranges from 1.0 to 41.2 in this study, mean = 8.5) measures a patch shape’s complexity by calculating how far it deviates from a circle or square of the same area. The patch cohesion index (cohesion, ranges from 84.9 to 100.0 in this study, mean = 97.8) measures the physical connectedness of the corresponding patch type. Patch cohesion increases as the patch type become more clumped or aggregated in its distribution, in other words, more physically connected. Therefore, higher index values of the LPI, shape, and cohesion mean larger greenspaces, more complex patch shapes, and more dense greenspaces. Compared with NDVI which could only reflect how dense an area of greenspace was, greenness structure indices were well-designed to show the exact shape, distribution, or connectivity of greenness. We calculated greenspace indices at the county level. China’s administrative divisions have three levels: provinces, counties, and townships, and there are 2846 county-level areas in China as the basic units of local administration. Greenspace indices were obtained from the outputs of the Advanced Land Observing Satellite (ALOS) based on the whole built environment of the county where each individual lived in 2008^36^ (Supplementary Methods). Considering the computing capability, we used 100 m × 100 m grid size in calculations.

Moreover, we used NDVI from the Moderate Resolution Imaging Spectro-Radiometer (MODIS) based on the longitude and latitude of each residential address as a measure of greenness surrounding the residence^37^. NDVI ranges from −1.0 to 1.0, with larger values indicating higher levels of vegetative density^38^. We deleted negative values, which represented blue space or water. We calculated contemporaneous NDVI at the individual’s residential address at the death date for individuals who had died /the last interview date for those who were alive and those lost to follow-up. There was no strong correlation between NDVI and greenness structures (Supplementary Table 3). Considering that there was only one specific and general area dimension of the relationship (positive, negative, or no relation) between NDVI values and frailty, separate greenness structures can capture different aspects of greenspace and describe the correlation between each aspect and frailty more accurately to contribute to precise urban green space planning in this case.

### Assessment of frailty

We used the frailty index (FI), which is defined as the ratio of the number of frailty items existing in an individual divided by the total self-reported items considered^39^, to measure frailty status according to the previous study^8,40^. FI had 39 self-reported items, including instrumental activities of daily living, functional limitations, activities of daily living, cognitive function, self-reported health status, interviewer-rated health status, mental health, auditory and visual ability, heart rhythm, and chronic diseases (Supplementary Table 2). We scored each term as 0 (without frailty status) or 1 (frailty status), except for item No.28 “number of serious illnesses in the past 2 years”, which was scored as 0 if the participant had no serious illness, 1 if the participant had one serious illness, or 2 if the participant reported two or more severe illnesses, such as stroke, cancer, and cataract. FI was equal to the number of reported items with frailty status divided by the total number of self-reported items. FI was a continuous variable and ranged from 0 to 1. A higher value indicated a higher degree of frailty. We also classified the continuous FI into two statuses as a binary variable: non-frail (FI ≤ 0.21) and frail (FI > 0.21), according to a previous study^41^. Changes of FI were the difference in FI scores measured between the last survey and the baseline, categorized as no change or decrease, and an increase.

### Covariates

The existing empirical literature has shown that the individual heterogeneity, socio-economic characteristics, and physical health status of older adults all affect the residential environment and frailty^42–44^. Therefore, we assessed a range of baseline characteristics to control the relevant individual, family, and regional differences. The study entrant year was the year when individuals entered the cohort. Age was divided into four groups, including the elderly (65–79), octogenarian (80–89), nonagenarian (90–99), and centenarian (100+). Literacy, annual household income, and BMI were defined according to the questionnaire of CLHLS. We dichotomized financial support to financial independence if the participants had their work and retirement wage, and financial dependence if they relied on other family members. Marital status (“currently married and living with spouse” or “other (separated, divorced, widowed, or never married)”), smoking status (“current smoker”, “former smoker” or “non-smoker”), alcohol consumption (“current drinker”, “former drinker” or “non-drinker”), physical activity (“current”, “former”, or “never”), residential location (city, town and rural area) were categorical variables. Smoking status, alcohol consumption, and physical activity were defined according to the questionnaire of CLHLS. For example, participants were asked, “do you exercise or not at present?” and/or “did you exercise or not in the past?”. We defined physical activity as “current exerciser” for participants who answered “Yes” to the first question, “former exerciser” for those who answered “No” to the first question and “Yes” to the second question, and “non-exerciser” for those who answered “No” to both two questions.

We divided the residence area into three levels according to the population, socio-economic development, and urbanization. Urban areas were committees and other districts that belong to the construction of district governments and municipal institutions in municipalities and unincorporated cities. Towns were committees and other districts associated with district governments and municipalities within the county and town construction outside of cities. Rural areas were places that are not part of towns and cities^45^. Based on the residential addresses of participants, we divided them into seven geographical regions to reflect differences in climate and dietary patterns: central China (Henan, Hubei, Hunan, and Jiangxi provinces), eastern China (Anhui, Fujian, Jiangsu, Shandong, Shanghai, and Zhejiang provinces), northeastern China (Heilongjiang, Jilin, and Liaoning provinces), northern China (Beijing, Inner Mongolia, Hebei, Shanxi and Tianjin provinces), northwestern China (Shaanxi, Ningxia, Xinjiang, Qinghai, and Gansu province), southern China (Guangdong, Guangxi, and Hainan provinces), and southwestern China (Chongqing, Yunnan, Guizhou, Xizang and Sichuan provinces). We calculated the three-year average (2006–2008) PM_2.5_, a kind of air-suspended mixture of both solid and liquid particles which have a diameter of 2.5 µm or smaller, to measure individual-level air pollution at 0.1° resolution based on the longitude and latitude of each residential address. PM_2.5_ particles are common air pollutants formed as a result of burning fuel and chemical reactions in the atmosphere. PM_2.5_ data was from a two-stage spatial statistical model developed by Ma et al., using the Moderate Resolution Imaging Spectroradiometer (MODIS) Collection 6 aerosol optical depth (AOD) and assimilated meteorology, land use data, and PM_2.5_ concentrations from China’s recently established ground monitoring network^46^.

### Statistical analysis

We hypothesized that a larger value of area-edge, shape, and proximity were protective factors for Chinese seniors’ frailty, and the strength of this protection varied among the subgroups of sex, age, literacy, urban or rural residential location, marital status, and changes of FI. First, a cross-sectional analysis was conducted using linear regression and logistic regression to assess the associations between residential greenness and frailty at baseline, adjusted for covariates. The linear regression was conducted to assess NDVI, greenspace structures, and continuous baseline FI scores; the logistic regression was conducted to calculate the odds ratio (OR) and 95% CIs to indicate associations between indices of greenspace structures and binary FI status. Considering the nonlinearity followed the process reported in the recent study^20,47,48^, NDVI, LPI, shape, and cohesion were further categorized into quartiles to do analysis. The lowest quartiles were the reference groups. Second, a longitudinal analysis was performed using the generalized estimating equations model of greenness structure indices and both continuous and binary FI among participants with follow-up. Additionally, we used contemporaneous NDVI classified into quartiles to reconfirm the association of exposure to overall greenness on frailty. All statistical analyses were conducted using R 3.6.3^49–51^.

### Sensitivity testing and subgroup analysis

We conducted sensitivity tests to examine the indices’ robustness^52^. We selected three other indices: edge density (ED) for area-edge, area-weighted mean fractal dimension index (FRAC) for shape, and percentage of like adjacencies (PLADJ) for proximity. ED equals the sum of the lengths of all greenspace edge segments per hectare. FRAC is another measure of shape complexity. PLADJ is calculated from the adjacency matrix, which measures the degree of aggregation of the focal patch type. Higher index values of the ED, FRAC, and PLADJ mean more greenspaces, more complex patch shape, and closer to greenspaces^33,53^. Besides, we did subgroup analyses based on sex, age, literacy, urban or rural residential location, marital status, and changes of FI from the end of the follow-up to the baseline.

### Reporting summary

Further information on research design is available in the Nature Research Reporting Summary linked to this article.

## Results

### Baseline characteristics

As shown in Supplementary Data 1, among 8776 individuals, 4135 (47.12%) participants were men, 5342 (60.87%) lived in rural regions, and 3295 (37.55%) were married and living with a spouse. The mean baseline LPI, Shape, Cohesion, and FI were 7.93, 8.11, 97.6, and 0.17. Compared with their counterparts, people living in bigger, more complex, and more tightly connected green areas tended to be female (LPI: 8.17 vs 8.12, Shape: 8.42 vs 8.15, Cohesion: 97.7 vs 97.6), literate (LPI: 8.49 vs 7.95, Shape: 8.35 vs 8.29, Cohesion: 97.9 vs 97.5), living in town (LPI: 9.88 vs 5.67, Shape: 9.54 vs 6.21, Cohesion: 98.0 vs 97.4), and not married or living with a spouse (LPI: 8.42 vs 7.53, Shape: 8.47 vs 7.94, Cohesion: 97.7 vs 97.6). People with healthier lifestyles such as keeping exercising (LPI: 8.35) or do not smoke (LPI: 8.43) had a higher value of LPI. The graphical mean values of baseline NDVI, LPI, Shape, and Cohesion of seven representative provinces of Eastern, Northeastern, Northern, Northwestern, Central, Southern, and Southwestern China are shown in Supplementary Fig. 1.

### The association between greenness structures and frailty

Supplementary Data 2 presents the association between greenness structure indices and frailty. From the cross-sectional analysis, we found that individuals who lived in greener areas with higher NDVI were less at risk of frailty (Coef = −0.014, 95%CI: −0.022 to −0.007, *P* < 0.001; OR = 0.794, 95%CI: 0.674–0.937, *P* < 0.01 in Q3). We also observed an association between a higher value of greenness structure indices and better frailty condition with a significant dose-response relationship in the quartiles group in the adjusted linear regression at baseline. Each 0.1-unit increase in LPI, Shape, and Cohesion was statistical significantly associated with a 0.026-point (95%CI: −0.033 to −0.019), 0.028-point (95%CI: −0.035 to −0.021), and 0.025-point (95%CI: −0.032 to −0.018) lower FI score in the fourth quartile. In the adjusted logistic regression, an increase in all greenness structure indices was associated with an OR less than 1 of frailty. Participants in the highest quartile had the lowest OR of LPI (0.676, 95%CI: 0.579–0.789, *P* < 0.001), Shape (0.650, 95%CI: 0.556–0.760, *P* < 0.001), and Cohesion (0.635, 95%CI: 0.541–0.744, *P* < 0.001). However, we did not find a similar negative association in the adjusted longitudinal GEE model where all results were insignificant.

### Sensitivity testing and subgroup analysis

**Fig. 2:**
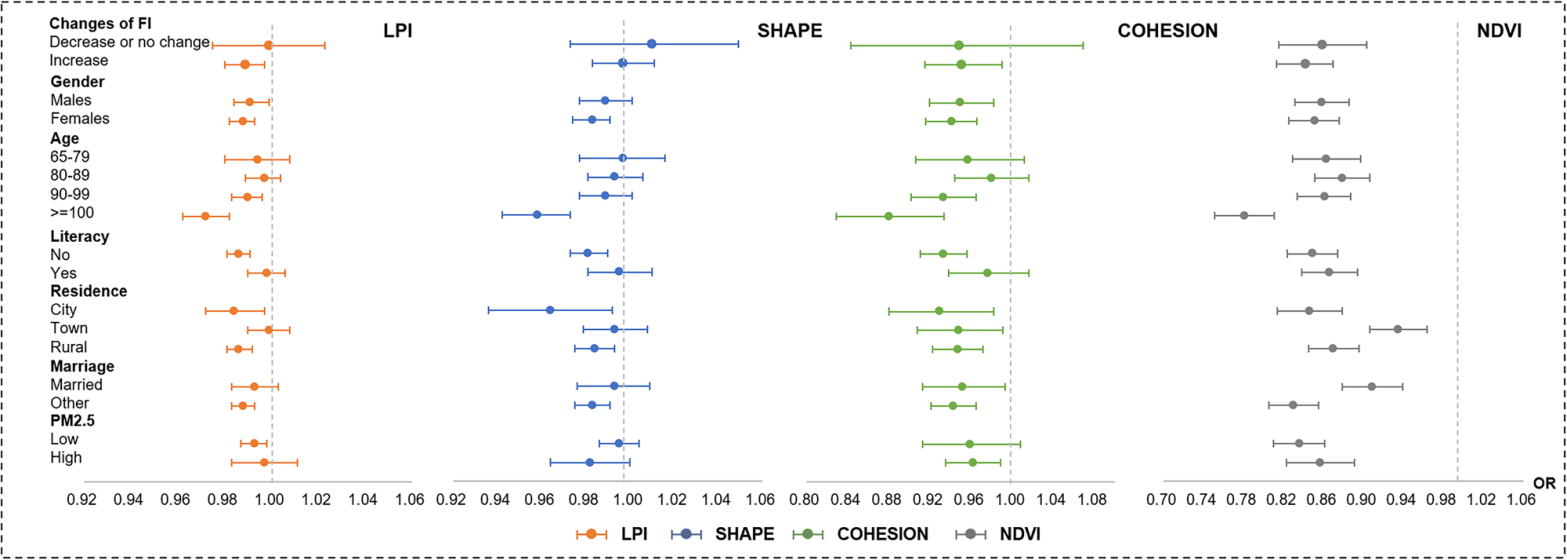
Subgroup analysis of the ORs (estimates with 95% CIs) of frailty indices of greenspace structures and NDVI according to changes of FI (*N* = 2855), sex, age, literacy, urban or rural residential location, marital status, and PM_2.5_ (*N* = 8776). Adjusted for age, sex, the study entrant year, marital status, geographic region, urban or rural residential location, literacy, annual household income, financial independence, BMI, smoking status, alcohol consumption, exercise status, three-year average, PM2.5. LPI: largest patch index. Shape: shape index. Cohesion: patch cohesion index. NDVI: normalized difference vegetation index. CI: confidence interval. OR: odds ratio.

The results of sensitivity analysis were basically consistent with the main model, indicating the specific indices type did not bias the results (Supplementary Data 3). Furthermore, the subgroup analyses showed similar findings in the cross-sectional analysis (Fig. 2, source data see Supplementary Data 4). We observed a more significant association among the females, centenarians, illiterate people, city residents, unmarried people. Compared with the participants with a lower or unchanged degree of frailty, those with a higher degree of frailty had lower ORs of LPI, Shape, and NDVI, and were noticeably benefited from Cohesion after adjustment.

## Discussion

In this prospective cohort study of older adults in China, we found a dose-response negative relationship for greenspace structures and frailty in cross-sectional analyses, indicating a larger area, more complex shape, more concentrated greenspaces, and greater proximity might reduce the risk of the frailty of older adults. However, we did not find a significant association in the longitudinal analysis partly because there were a large number of deaths and lost to follow-up after the baseline survey. Compared with these people, participants with follow-up surveys had better frailty at baseline (Supplementary Table 4), causing the protective effect of greenspace structure to became unobvious. There are, however, other possible explanations. For example, the choice of the statistical model is not optimal, deletion of non-responses, confounders might be influenced by time, etc. Therefore, further research should be undertaken to investigate the related issues and verify the results.

Previous research has established that older people living in neighborhoods with a higher percentage of greenspaces (larger NDVI) had a higher likelihood of improvement in frailty status^7,8^, and our study is in line with those of previous studies. Greenspace is an important part of neighborhoods that support healthy and active lifestyles. Increasing greenspace area and appropriate planning around the residency may promote cognitive function and psychological states, which then has an influence on frailty risk^7^. Besides, our findings further increase understanding of how greenness structures specifically supplement NDVI’s practical implications on urban greenspace planning by giving evidence on the possibility that green structures improve frailty through different paths. First, large area-edge and good proximity can improve frailty by increasing opportunities for physical exercise, which is a mediator of the relationship between greenspaces and frailty transitions by improving physical, cognitive, and psychological function^7,54^. Meanwhile, most people prefer to enter green areas with a larger size, which can afford plenty of room to do diverse health-related activities^55^. Good greenness connectivity maintained by green ecological corridors also provides opportunities for physical activities^56^. However, these results must be interpreted with caution because of the bidirectional influences of frailty and residential greenness. For example, older adults who are less frail are more likely to actively choose to live in a greener place because they have more needs for certain physical activities or gardening^57^. Second, there is evidence that greenspace may influence health by directly promoting cognitive functions and well-being, strongly related to the onset of frailty^7,37,58^. Complex shapes of greenspaces diversify public spaces and enhance neighborhood satisfaction^59^, and interconnecting greenspaces play a critical role in providing comfortable environments^60^, which could increase memory, attention^61^, and mental health^62^. Third, exposure to air pollution has been linked to respiratory diseases, and maybe contributory to frailty^63^. Minimized fragmentation and increased the largest patch percentage of green structure could lower the mortality of pneumonia and chronic lower respiratory diseases by and the mediation effects through reducing air pollutants^64^.

The present study observes that greenspace indices’ protective effects were more evident on city residents, people who were unmarried and not living with a spouse, and with an increased degree of frailty. A study in the Netherlands reported the significant association between greenspaces and different perceived general health among different levels of urbanization^57^. China has witnessed rapid urbanization widening the gap of unequal landscaping plans in urban and rural areas^65,66^. Another possible explanation for urban-rural differences is that in rural areas, lower socioeconomic status, high risks of infectious diseases, and lack of universal health coverage undermine the positive role of greenspaces^67^. Additionally, it might also be due to the urban-rural difference in FI at baseline (urban 0.18 vs. rural 0.16). Besides, previous research has established that marriage could cause a difference in frailty. In our study, individuals who were unmarried and not living with a spouse were frailer than their counterparts (0.20 vs. 0.11). This can be explained by the relationship between frailty and psychosocial factors. Spouses might bring positive emotions which relate to individual health^68^. Therefore, if greenspaces can provide widowed older adults with another form of positive emotions, it might be able to play a compensatory role in mental health. We also find that compared with older adults who still have steady or even better frailty, participants with an increased degree of frailty had a higher possibility to be protected by large and tightly connected greenspaces. If the area and distribution of greenness can indeed delay the frailty process, greenspace planning can become a tool for healthy aging.

This study had several limitations. First, 5921 individuals did not have FI scores in 2014 because of death or lost follow-up, which might affect the longitudinal analysis accuracy. We only observed significant results in the cross-sectional analysis, which also were subject to biases. Second, we only used greenness structures indices in 2008. Future studies might use data in different periods to substantiate our analyses. Third, we could not get information about the specific types of vegetation, the time participants spent in the greenspaces, and participants’ activity patterns via satellite. Fourth, there may be some confounders and potential mediators (e.g., neighborhood safety, social network) that we could not account for in the models. Nevertheless, this study involved notable strengths. First, as far as we know, our study is the first on the association between greenspace structures and frailty in older adults from a cohort that covers the majority of regions in China. We also used high-quality geographic information system data to quantify greenspace structures and included a wide range of demographic and socioeconomic variables to control potential confounding. This research provides insights for the comparison of the effects of NDVI and greenspace structures on frailty. A further study could assess the specific mechanism of the combined impact of NDVI and structures on health.

## Supplementary information

Reporting Summary

Supplementary Data 1

Supplementary Data 2

Supplementary Data 3

Supplementary Data 4

Supplementary Information

Description of Additional Supplementary Files

## Data Availability

The epidemiological data on the CLHLS cohort that support the findings of this study are available in Peking University Open Research Data with the identifier [10.18170/DVN/UWS2LR]^69^. The greenspace data that support the findings of this study are publicly available from Advanced Land Observing Satellite, https://www.eorc.jaxa.jp/ALOS/en/index_e.htm. Source data for Fig. 2 is available as a Supplementary Data file.

## Code availability

R packages and software associated with these analyses is available at https://cran.r-project.org/web/packages/gee/index.html and https://www.umass.edu/landeco/research/fragstats/fragstats.html. The R codes used in this study are freely available at https://github.com/yilijiaer/Greenness_FI^70^.
