# Supplementary material for: Association between residential greenspace structures and frailty in a cohort of older Chinese adults
Source: Commun Med (Lond). 2022 Apr 20;2:43. doi: 10.1038/s43856-022-00093-9 (PMC9053290; doi:10.1038/s43856-022-00093-9)
Supplement: Supplementary file 1 — Reporting Summary [file 43856_2022_93_MOESM1_ESM.pdf]

nature research

Corresponding author(s): John S. Ji, Chih-da Wu

Last updated by author(s): Feb 26, 2022

Reporting Summary

Nature Research wishes to improve the reproducibility of the work that we publish. This form provides structure for consistency and transparency in reporting. For further information on Nature Research policies, see our Editorial Policies and the Editorial Policy Checklist.

Statistics

For all statistical analyses, confirm that the following items are present in the figure legend, table legend, main text, or Methods section.

n/a	Confirmed	
☐	☒	The exact sample size (n) for each experimental group/condition, given as a discrete number and unit of measurement
☐	☒	A statement on whether measurements were taken from distinct samples or whether the same sample was measured repeatedly
☐	☒	The statistical test(s) used AND whether they are one- or two-sided<br>*Only common tests should be described solely by name; describe more complex techniques in the Methods section.*
☐	☒	A description of all covariates tested
☐	☒	A description of any assumptions or corrections, such as tests of normality and adjustment for multiple comparisons
☐	☒	A full description of the statistical parameters including central tendency (e.g. means) or other basic estimates (e.g. regression coefficient) AND variation (e.g. standard deviation) or associated estimates of uncertainty (e.g. confidence intervals)
☐	☒	For null hypothesis testing, the test statistic (e.g. F, t, r) with confidence intervals, effect sizes, degrees of freedom and P value noted<br>*Give P values as exact values whenever suitable.*
☒	☐	For Bayesian analysis, information on the choice of priors and Markov chain Monte Carlo settings
☒	☐	For hierarchical and complex designs, identification of the appropriate level for tests and full reporting of outcomes
☐	☒	Estimates of effect sizes (e.g. Cohen's d, Pearson's r), indicating how they were calculated

Our web collection on statistics for biologists contains articles on many of the points above.

Software and code

Policy information about availability of computer code

Data collection	The epidemiological data on the CLHLS cohort that support the findings of this study are publicly available in Peking University Open Research Data with the identifier [https://doi.org/10.18170/DVN/UWS2LR]. The greenspace data that support the findings of this study are publicly available from Advanced Land Observing Satellite, https://www.eorc.jaxa.jp/ALOS/en/index_e.htm. Source data for Fig 2 is available as a Supplementary Data file.
Data analysis	R packages and software associated with these analyses is available at https://cran.r-project.org/web/packages/gee/index.html and https://www.umass.edu/landeco/research/fragstats/fragstats.html. The R codes used in this study are freely available at https://github.com/yilijiaer/Greenness_FI (https://zenodo.org/badge/latestdoi/414160315).

For manuscripts utilizing custom algorithms or software that are central to the research but not yet described in published literature, software must be made available to editors and reviewers. We strongly encourage code deposition in a community repository (e.g. GitHub). See the Nature Research guidelines for submitting code & software for further information.

Data

Policy information about availability of data

All manuscripts must include a data availability statement. This statement should provide the following information, where applicable:

- Accession codes, unique identifiers, or web links for publicly available datasets
- A list of figures that have associated raw data
- A description of any restrictions on data availability

The epidemiological data on the CLHLS cohort that support the findings of this study are publicly available in Peking University Open Research Data with the identifier [https://doi.org/10.18170/DVN/UWS2LR]. The greenspace data that support the findings of this study are publicly available from Advanced Land Observing Satellite, https://www.eorc.jaxa.jp/ALOS/en/index_e.htm. Source data for Fig 2 is available as a Supplementary Data file.

Field-specific reporting

Please select the one below that is the best fit for your research. If you are not sure, read the appropriate sections before making your selection.

☒ Life sciences ☐ Behavioural & social sciences ☐ Ecological, evolutionary & environmental sciences

For a reference copy of the document with all sections, see nature.com/documents/nr-reporting-summary-flat.pdf

Life sciences study design

All studies must disclose on these points even when the disclosure is negative.

Sample size	This is a longitudinal cohort study.We included older adults from the 2008-2014 waves of the China Longitudinal Healthy Longevity Survey (CLHLS), which consisted of 16,072 individuals received baseline interviews in 2008/09, and follow-ups in 2012 and 2014. About 50% of counties/county-level cities/districts were randomly selected from 22 provinces/municipalities/autonomous regions; All surviving centenarians who volunteered to participate in the survey were interviewed in households in these areas; As far as possible, one person aged 80-89 and one person aged 90-99 should be interviewed nearby. These two interviewed people were determined after randomly selecting age and gender according to the number of centenarians. The basic idea of selecting samples in this way is as follows: the number of respondents of 80-89 years old and 90-99 years old respectively is about the same as that of centenarians, and the number of respondents of 80-99 years old male and female respectively is about the same. Since 2002, a subsample of 65-79 year olds has been added, and the sample of 65-79 year olds is the same as the sample of 80-99 year olds mentioned above.In the follow-up survey, the deceased and lost to follow-up of the elderly according to the same gender, the same age principle of the nearest replacement sample.
Data exclusions	We excluded individuals who had missing demographic characters (N=3,073), frailty index (N=4,215), and NDVI (N=8) at baseline year. There was no significant difference between baseline characters of excluded individuals and the overall sample. Final sample size was 8,776 at baseline year.
Replication	We conducted sensitivity tests to examine the indices' robustness. We selected different greenness structure indices and did four subgroup analyses based on sex, age, literacy, urban or rural residential location, and marital status.
Randomization	One person aged 80-89 and one person aged 90-99 should be interviewed nearby centenarians. These two interviewed people were determined after randomly selecting age and gender according to the number of centenarians. The basic idea of selecting samples in this way is as follows: the number of respondents of 80-89 years old and 90-99 years old respectively is about the same as that of centenarians, and the number of respondents of 80-99 years old male and female respectively is about the same. Covariates: age, sex, the study entrant year, marital status, geographic region, urban or rural residential location, literacy, annual household income, BMI, smoking status, alcohol consumption, exercise status, three-year average, PM2.5.
Blinding	Blinding was not relevant to this study because it is an observational cohort study.

Behavioural & social sciences study design

All studies must disclose on these points even when the disclosure is negative.

Study description	*Briefly describe the study type including whether data are quantitative, qualitative, or mixed-methods (e.g. qualitative cross-sectional, quantitative experimental, mixed-methods case study).*
Research sample	*State the research sample (e.g. Harvard university undergraduates, villagers in rural India) and provide relevant demographic information (e.g. age, sex) and indicate whether the sample is representative. Provide a rationale for the study sample chosen. For studies involving existing datasets, please describe the dataset and source.*
Sampling strategy	*Describe the sampling procedure (e.g. random, snowball, stratified, convenience). Describe the statistical methods that were used to predetermine sample size OR if no sample-size calculation was performed, describe how sample sizes were chosen and provide a rationale for why these sample sizes are sufficient. For qualitative data, please indicate whether data saturation was considered, and what criteria were used to decide that no further sampling was needed.*
Data collection	*Provide details about the data collection procedure, including the instruments or devices used to record the data (e.g. pen and paper, computer, eye tracker, video or audio equipment) whether anyone was present besides the participant(s) and the researcher, and whether the researcher was blind to experimental condition and/or the study hypothesis during data collection.*
Timing	*Indicate the start and stop dates of data collection. If there is a gap between collection periods, state the dates for each sample cohort.*
Data exclusions	*If no data were excluded from the analyses, state so OR if data were excluded, provide the exact number of exclusions and the rationale behind them, indicating whether exclusion criteria were pre-established.*
Non-participation	*State how many participants dropped out/declined participation and the reason(s) given OR provide response rate OR state that no participants dropped out/declined participation.*

Randomization | *If participants were not allocated into experimental groups, state so OR describe how participants were allocated to groups, and if allocation was not random, describe how covariates were controlled.*

Ecological, evolutionary & environmental sciences study design

All studies must disclose on these points even when the disclosure is negative.

Study description | This is a longitudinal cohort study.

Research sample | We included older adults from the 2008-2014 waves of the China Longitudinal Healthy Longevity Survey (CLHLS), which consisted of 16,072 individuals received baseline interviews in 2008/09, and follow-ups in 2012 and 2014.

Sampling strategy | (1) About 50% of counties/county-level cities/districts were randomly selected from 22 provinces/municipalities/autonomous regions;
(2) All surviving centenarians who volunteered to participate in the survey were interviewed in households in these areas;
(3) As far as possible, one person aged 80-89 and one person aged 90-99 should be interviewed nearby. These two interviewed people were determined after randomly selecting age and gender according to the number of centenarians. The basic idea of selecting samples in this way is as follows: the number of respondents of 80-89 years old and 90-99 years old respectively is about the same as that of centenarians, and the number of respondents of 80-99 years old male and female respectively is about the same. Since 2002, a subsample of 65-79 year olds has been added, and the sample of 65-79 year olds is the same as the sample of 80-99 year olds mentioned above.In the follow-up survey, the deceased and lost to follow-up of the elderly according to the same gender, the same age principle of the nearest replacement sample.

Data collection | Data Collection Organizations: Peking University Center for Healthy Aging and Family Studies (CHAFS) and China Mainland Information Group (MIG)

Timing and spatial scale | From 2008 to 2014.

Data exclusions | We excluded individuals who had missing demographic characters (N=3,073), frailty index (N=4,215), and NDVI (N=8) at baseline year. There was no significant difference between baseline characters of excluded individuals and the overall sample. Final sample size was 8,776 at baseline year.

Reproducibility | We conducted sensitivity tests to examine the indices' robustness. We selected different greenness structure indices and did four subgroup analyses based on sex, age, literacy, urban or rural residential location, and marital status.

Randomization | One person aged 80-89 and one person aged 90-99 should be interviewed nearby centenarians. These two interviewed people were determined after randomly selecting age and gender according to the number of centenarians. The basic idea of selecting samples in this way is as follows: the number of respondents of 80-89 years old and 90-99 years old respectively is about the same as that of centenarians, and the number of respondents of 80-99 years old male and female respectively is about the same. Covariates: age, sex, the study entrant year, marital status, geographic region, urban or rural residential location, literacy, annual household income, BMI, smoking status, alcohol consumption, exercise status, three-year average, PM2.5.

Blinding | The study was not blinded because it was observational, not a intervention study.

Did the study involve field work? ☐ Yes ☒ No

Field work, collection and transport

Field conditions | *Describe the study conditions for field work, providing relevant parameters (e.g. temperature, rainfall).*

Location | *State the location of the sampling or experiment, providing relevant parameters (e.g. latitude and longitude, elevation, water depth).*

Access & import/export | *Describe the efforts you have made to access habitats and to collect and import/export your samples in a responsible manner and in compliance with local, national and international laws, noting any permits that were obtained (give the name of the issuing authority, the date of issue, and any identifying information).*

Disturbance | *Describe any disturbance caused by the study and how it was minimized.*

Reporting for specific materials, systems and methods

We require information from authors about some types of materials, experimental systems and methods used in many studies. Here, indicate whether each material, system or method listed is relevant to your study. If you are not sure if a list item applies to your research, read the appropriate section before selecting a response.

Materials & experimental systems

n/a	Involved in the study
☒	☐ Antibodies
☒	☐ Eukaryotic cell lines
☒	☐ Palaeontology and archaeology
☒	☐ Animals and other organisms
☐	☒ Human research participants
☒	☐ Clinical data
☒	☐ Dual use research of concern

Methods

n/a	Involved in the study
☒	☐ ChIP-seq
☒	☐ Flow cytometry
☒	☐ MRI-based neuroimaging

Antibodies

Antibodies used — *Describe all antibodies used in the study; as applicable, provide supplier name, catalog number, clone name, and lot number.*

Validation — *Describe the validation of each primary antibody for the species and application, noting any validation statements on the manufacturer's website, relevant citations, antibody profiles in online databases, or data provided in the manuscript.*

Eukaryotic cell lines

Policy information about cell lines

Cell line source(s) — *State the source of each cell line used.*

Authentication — *Describe the authentication procedures for each cell line used OR declare that none of the cell lines used were authenticated.*

Mycoplasma contamination — *Confirm that all cell lines tested negative for mycoplasma contamination OR describe the results of the testing for mycoplasma contamination OR declare that the cell lines were not tested for mycoplasma contamination.*

Commonly misidentified lines (See ICLAC register) — *Name any commonly misidentified cell lines used in the study and provide a rationale for their use.*

Palaeontology and Archaeology

Specimen provenance — *Provide provenance information for specimens and describe permits that were obtained for the work (including the name of the issuing authority, the date of issue, and any identifying information).*

Specimen deposition — *Indicate where the specimens have been deposited to permit free access by other researchers.*

Dating methods — *If new dates are provided, describe how they were obtained (e.g. collection, storage, sample pretreatment and measurement), where they were obtained (i.e. lab name), the calibration program and the protocol for quality assurance OR state that no new dates are provided.*

☐ Tick this box to confirm that the raw and calibrated dates are available in the paper or in Supplementary Information.

Ethics oversight — *Identify the organization(s) that approved or provided guidance on the study protocol, OR state that no ethical approval or guidance was required and explain why not.*

Note that full information on the approval of the study protocol must also be provided in the manuscript.

Animals and other organisms

Policy information about studies involving animals; ARRIVE guidelines recommended for reporting animal research

Laboratory animals — *For laboratory animals, report species, strain, sex and age OR state that the study did not involve laboratory animals.*

Wild animals — *Provide details on animals observed in or captured in the field; report species, sex and age where possible. Describe how animals were caught and transported and what happened to captive animals after the study (if killed, explain why and describe method; if released, say where and when) OR state that the study did not involve wild animals.*

Field-collected samples — *For laboratory work with field-collected samples, describe all relevant parameters such as housing, maintenance, temperature, photoperiod and end-of-experiment protocol OR state that the study did not involve samples collected from the field.*

Ethics oversight — *Identify the organization(s) that approved or provided guidance on the study protocol, OR state that no ethical approval or guidance was required and explain why not.*

Note that full information on the approval of the study protocol must also be provided in the manuscript.

Human research participants

Policy information about studies involving human research participants

Population characteristics	Among 8,776 individuals in this study, 4,135 (47.12%) participants were men, 5,342 (60.87%) lived in rural regions, and 3,295 (37.55%) were married and living with a spouse.
Recruitment	The CLHLS was a national survey for investigating the determinants of healthy longevity among the older Chinese population in 22 provinces. The survey has drawn areas from a population base of 1.1 billion people, representing 85 percent of China's total population. Investigators interviewed individuals about socioeconomic characteristics, lifestyle, physical capacity, cognitive function, and psychological status. The sampling design of this survey adopts multi-stage unequal proportion target random sampling method. This study used the 2008-2014 data which consisted of 16,072 individuals received baseline interviews in 2008/09, and follow-ups in 2012 and 2014.
Ethics oversight	CLHLS was approved by the medical ethics committee of PKU. The IRB approval and other information were described in the published cohort profile (https://opendata.pku.edu.cn/dataset.xhtml?persistentId=doi:10.18170/DVN/UWS2LR).

Note that full information on the approval of the study protocol must also be provided in the manuscript.

Clinical data

Policy information about clinical studies

All manuscripts should comply with the ICMJE guidelines for publication of clinical research and a completed CONSORT checklist must be included with all submissions.

Clinical trial registration	*Provide the trial registration number from ClinicalTrials.gov or an equivalent agency.*
Study protocol	*Note where the full trial protocol can be accessed OR if not available, explain why.*
Data collection	*Describe the settings and locales of data collection, noting the time periods of recruitment and data collection.*
Outcomes	*Describe how you pre-defined primary and secondary outcome measures and how you assessed these measures.*

Dual use research of concern

Policy information about dual use research of concern

Hazards

Could the accidental, deliberate or reckless misuse of agents or technologies generated in the work, or the application of information presented in the manuscript, pose a threat to:

No	Yes	
☐	☐	Public health
☐	☐	National security
☐	☐	Crops and/or livestock
☐	☐	Ecosystems
☐	☐	Any other significant area

Experiments of concern

Does the work involve any of these experiments of concern:

No	Yes	
☐	☐	Demonstrate how to render a vaccine ineffective
☐	☐	Confer resistance to therapeutically useful antibiotics or antiviral agents
☐	☐	Enhance the virulence of a pathogen or render a nonpathogen virulent
☐	☐	Increase transmissibility of a pathogen
☐	☐	Alter the host range of a pathogen
☐	☐	Enable evasion of diagnostic/detection modalities
☐	☐	Enable the weaponization of a biological agent or toxin
☐	☐	Any other potentially harmful combination of experiments and agents

ChIP-seq

Data deposition

- [ ] Confirm that both raw and final processed data have been deposited in a public database such as GEO.
- [ ] Confirm that you have deposited or provided access to graph files (e.g. BED files) for the called peaks.

Data access links<br>*May remain private before publication.*	*For "Initial submission" or "Revised version" documents, provide reviewer access links. For your "Final submission" document, provide a link to the deposited data.*
Files in database submission	*Provide a list of all files available in the database submission.*
Genome browser session<br>(e.g. UCSC)	*Provide a link to an anonymized genome browser session for "Initial submission" and "Revised version" documents only, to enable peer review. Write "no longer applicable" for "Final submission" documents.*

Methodology

Replicates	*Describe the experimental replicates, specifying number, type and replicate agreement.*
Sequencing depth	*Describe the sequencing depth for each experiment, providing the total number of reads, uniquely mapped reads, length of reads and whether they were paired- or single-end.*
Antibodies	*Describe the antibodies used for the ChIP-seq experiments; as applicable, provide supplier name, catalog number, clone name, and lot number.*
Peak calling parameters	*Specify the command line program and parameters used for read mapping and peak calling, including the ChIP, control and index files used.*
Data quality	*Describe the methods used to ensure data quality in full detail, including how many peaks are at FDR 5% and above 5-fold enrichment.*
Software	*Describe the software used to collect and analyze the ChIP-seq data. For custom code that has been deposited into a community repository, provide accession details.*

Flow Cytometry

Plots

Confirm that:

- [ ] The axis labels state the marker and fluorochrome used (e.g. CD4-FITC).
- [ ] The axis scales are clearly visible. Include numbers along axes only for bottom left plot of group (a 'group' is an analysis of identical markers).
- [ ] All plots are contour plots with outliers or pseudocolor plots.
- [ ] A numerical value for number of cells or percentage (with statistics) is provided.

Methodology

Sample preparation	*Describe the sample preparation, detailing the biological source of the cells and any tissue processing steps used.*
Instrument	*Identify the instrument used for data collection, specifying make and model number.*
Software	*Describe the software used to collect and analyze the flow cytometry data. For custom code that has been deposited into a community repository, provide accession details.*
Cell population abundance	*Describe the abundance of the relevant cell populations within post-sort fractions, providing details on the purity of the samples and how it was determined.*
Gating strategy	*Describe the gating strategy used for all relevant experiments, specifying the preliminary FSC/SSC gates of the starting cell population, indicating where boundaries between "positive" and "negative" staining cell populations are defined.*

- [ ] Tick this box to confirm that a figure exemplifying the gating strategy is provided in the Supplementary Information.

Magnetic resonance imaging

Experimental design

Design type	*Indicate task or resting state; event-related or block design.*

Design specifications	*Specify the number of blocks, trials or experimental units per session and/or subject, and specify the length of each trial or block (if trials are blocked) and interval between trials.*
Behavioral performance measures	*State number and/or type of variables recorded (e.g. correct button press, response time) and what statistics were used to establish that the subjects were performing the task as expected (e.g. mean, range, and/or standard deviation across subjects).*

Acquisition

Imaging type(s)	*Specify: functional, structural, diffusion, perfusion.*
Field strength	*Specify in Tesla*
Sequence & imaging parameters	*Specify the pulse sequence type (gradient echo, spin echo, etc.), imaging type (EPI, spiral, etc.), field of view, matrix size, slice thickness, orientation and TE/TR/flip angle.*
Area of acquisition	*State whether a whole brain scan was used OR define the area of acquisition, describing how the region was determined.*

Diffusion MRI ☐ Used ☐ Not used

Preprocessing

Preprocessing software	*Provide detail on software version and revision number and on specific parameters (model/functions, brain extraction, segmentation, smoothing kernel size, etc.).*
Normalization	*If data were normalized/standardized, describe the approach(es): specify linear or non-linear and define image types used for transformation OR indicate that data were not normalized and explain rationale for lack of normalization.*
Normalization template	*Describe the template used for normalization/transformation, specifying subject space or group standardized space (e.g. original Talairach, MNI305, ICBM152) OR indicate that the data were not normalized.*
Noise and artifact removal	*Describe your procedure(s) for artifact and structured noise removal, specifying motion parameters, tissue signals and physiological signals (heart rate, respiration).*
Volume censoring	*Define your software and/or method and criteria for volume censoring, and state the extent of such censoring.*

Statistical modeling & inference

Model type and settings	*Specify type (mass univariate, multivariate, RSA, predictive, etc.) and describe essential details of the model at the first and second levels (e.g. fixed, random or mixed effects; drift or auto-correlation).*
Effect(s) tested	*Define precise effect in terms of the task or stimulus conditions instead of psychological concepts and indicate whether ANOVA or factorial designs were used.*

Specify type of analysis: ☐ Whole brain ☐ ROI-based ☐ Both

Statistic type for inference (See Eklund et al. 2016)	*Specify voxel-wise or cluster-wise and report all relevant parameters for cluster-wise methods.*
Correction	*Describe the type of correction and how it is obtained for multiple comparisons (e.g. FWE, FDR, permutation or Monte Carlo).*

Models & analysis

n/a	Involved in the study
☐	☐ Functional and/or effective connectivity
☐	☐ Graph analysis
☐	☐ Multivariate modeling or predictive analysis

Functional and/or effective connectivity	*Report the measures of dependence used and the model details (e.g. Pearson correlation, partial correlation, mutual information).*
Graph analysis	*Report the dependent variable and connectivity measure, specifying weighted graph or binarized graph, subject- or group-level, and the global and/or node summaries used (e.g. clustering coefficient, efficiency, etc.).*
Multivariate modeling and predictive analysis	*Specify independent variables, features extraction and dimension reduction, model, training and evaluation metrics.*
