# Supplementary material for: Association between residential greenspace structures and frailty in a cohort of older Chinese adults
Source: Commun Med (Lond). 2022 Apr 20;2:43. doi: 10.1038/s43856-022-00093-9 (PMC9053290; doi:10.1038/s43856-022-00093-9)
Supplement: Supplementary file 6 — Supplementary Information [file 43856_2022_93_MOESM6_ESM.pdf]

Supplementary Table 1. Formula and descriptions for the indices of greenspace structures.

Indices	Formula	Description
**Area-Edge**		
Largest Patch Index (LPI)	$\frac{\max_{j=1}^{a}(a_{ij})}{A}(100)$	LPI equals the area (m2) of the largest patch of the corresponding patch type divided by total landscape area (m2), multiplied by 100 (to convert to a percentage).
Edge Density (ED)	$\frac{E}{A}(10{,}000)$	ED equals the sum of the lengths (m) of all edge segments in the landscape, divided by the total landscape area (m^2), multiplied by 10,000.
**Shape**		
Shape Index (Shape)	$\frac{\sum_{i=1}^{m}\sum_{j=1}^{n}\frac{p_{ij}}{min\ p_{ij}}}{N}$	Shape Index equals the sum, across all patches in the landscape, of the SHAPE value for each patch, divided by the total number of patches.
Area-weighted mean fractal dimension index (FRAC)	$k * A^{D/2}$	Fractal analysis usually is applied to the entire landscape mosaic using the perimeter-area relationship. Area-weighted mean fractal dimension index was calculated by weighting patches according to their size.
**Proximity**		
Patch Cohesion Index (Cohesion)	$[1-\frac{\sum_{i=1}^{n} p_{ij}}{\sum_{i=1}^{n} p_{ij}\sqrt{a_{ij}}}][1-\frac{1}{\sqrt{A}}]^{-1}\cdot(100)$	COHESION equals 1 minus the sum of patch perimeter divided by the sum of patch perimeter times the square root of patch area for patches of the corresponding patch type, divided by 1 minus 1 over the square root of the total number of cells in the landscape, multiplied by 100 to convert to a percentage.
Percentage of Like Adjacencies (PLADJ)	$\left[\frac{g_{ij}}{\sum_{k=1}^{m} g_{ik}}\right](100)$	PLADJ equals the number of like adjacencies involving the focal class, divided by the total number of cell adjacencies involving the focal class; multiplied by 100,

Supplementary Table 2. The list of items included in calculating the FI.

No.	Items
1	IADL: Unable to visit neighbors by oneself
2	IADL: Unable to shop by oneself if necessary
3	IADL: Unable to cook meals by oneself if necessary
4	IADL: Unable to wash clothing by oneself
5	IADL: Unable to walk continuously for 1 km
6	IADL: Unable to lift a weight of 5 kg (such as a heavy bag of groceries)
7	IADL: Unable to continuously crouch and stand up 3 times
8	IADL: Unable to use public transportation
9	Functional limitations: Unable to put hand behind neck
10	Functional limitations: Unable to put hand behind lower back
11	Functional limitations: Unable to raise arm upright
12	Functional limitations: Unable to stand up from sitting in a chair
13	Functional limitations: Unable to pick up a book from the floor
14	ADL: Needs assistance bathing
15	ADL: Needs assistance dressing
16	ADL: Needs assistance toileting
17	ADL: Needs assistance in indoor transferring
18	ADL: Needs assistance eating
19	ADL: Incontinence
20	Cognitively impaired (based on the MMSE)
21	Poor self-rated health
22	Health worsened in the past year
23	Poor interviewer-rated health
24	Hearing loss
25	Vision loss
26	Abnormal heart rhythm
27	Symptom of psychological distress (based on loneliness, fearfulness)
28	Number of serious illnesses in the past 2 years
29	Suffering from hypertension
30	Suffering from diabetes
31	Suffering from tuberculosis
32	Suffering from heart disease
33	Suffering from stroke/cerebrovascular disease
34	Suffering from bronchitis
35	Suffering from cancer
36	Suffering from arthritis
37	Suffering from bedsores
38	Suffering from gastric or duodenal ulcers
39	Suffering from Parkinson's disease

IADLs, instrumental activities of daily living; ADL, activities of daily living. Item no. 28 was assigned a value of 2.

Supplementary Table 3. Pearson correlation matrix between greenspace structures and NDVI.

Variable	LPI	ED	Shape	Frac	Cohesion	PLADJ	NDVI
**LPI**	1						
**ED**	.597**	1					
**Shape**	.669**	.631**	1				
**Frac**	.678**	.690**	.698**	1			
**Cohesion**	.508**	.437**	.560**	.621**	1		
**PLADJ**	.668**	.428**	.570**	.612**	.663**	1	
**NDVI**	.047*	.196**	.099**	.062**	-.088**	-.172**	1

** *P*<0.01, * *P*<0.05. LPI: largest patch index. ED: edge density. Shape: shape index. Frac: fractal dimension index. Cohesion: patch cohesion index. PLADJ: percentage of like adjacencies. NDVI: normalized difference vegetation index.

Supplementary Table 4. Baseline NDVI, LPI, Shape, Cohesion, and FI for the participants with or without follow-up surveys.

	**All Participants (N = 8,776)**	**Participants with Follow-up Surveys (N = 2,855)**	**Participants Without Follow-up Surveys**		
			All (N = 5,921)	Death (N = 4,318)	Lost to Follow-up (N = 1,603)
**Baseline NDVI, mean ± SD**	0.41 (0.20)	0.44 (0.19)	0.40 (0.21)	0.39 (0.20)	0.45 (0.20)
**Baseline LPI, mean ± SD**	7.93 (12.30)	8.64 (12.58)	8.85 (13.06)	9.43 (13.37)	7.36 (12.09)
**Baseline Shape, mean ± SD**	8.11 (7.58)	8.47 (7.49)	8.72 (8.05)	8.98 (8.15)	8.05 (7.78)
**Baseline Cohesion, mean ± SD**	97.6 (2.69)	97.78 (2.62)	97.79 (2.63)	97.84 (2.67)	97.67 (2.53)
**Baseline FI, mean ± SD**	0.17 (0.15)	0.10 (0.09)	0.20 (0.15)	0.22 (0.16)	0.12 (0.12)

NDVI: normalized difference vegetation index. LPI: largest patch index. Shape: shape index. Cohesion: patch cohesion index. FI: frailty index.

Supplementary Data 3. Sensitivity analysis of frailty and other indices of greenspace structures in China.

Supplementary Figure 1. The mean value of 2008-baseline NDVI, LPI, Shape, and Cohesion of seven representative provinces of Eastern, Northeastern, Northern, Northwestern, Central, Southern, and Southwestern China.

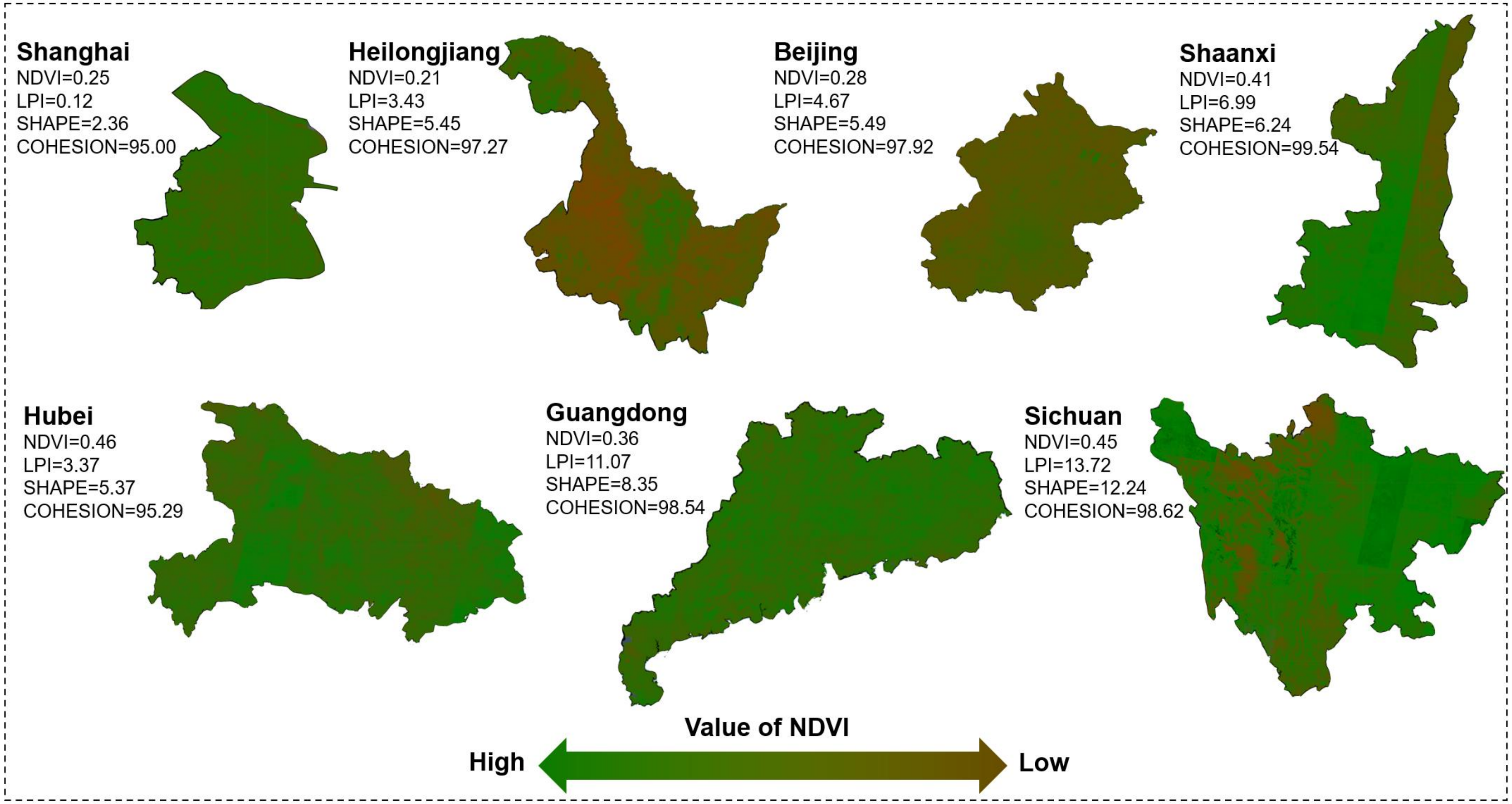

LPI: largest patch index. Shape: shape index. Cohesion: patch cohesion index.

Supplementary Methods. Official overview of the ALOS dataset

Global 25m resolutions PALSAR-2/PALSAR mosaic and forest/non-forest map are free and open dataset generated by applying JAXA's powerful processing and sophisticated analysis method/techniques to a lot of images obtained with Japanese L-band Synthetic Aperture Radars (PALSAR and PALSAR-2) on Advanced Land Observing Satellite (ALOS) and Advanced Land Observing Satellite-2 (ALOS-2). For understanding and responding to global environmental changes such as global warming and loss of biodiversity, timely assessment of deforestation and forest degradation is essential. Global monitoring with satellite remote sensing is one of the most effective approaches to detect land surface changes because satellites can provide wall-to-wall images covering wide areas periodically. L-band Synthetic Aperture Radars (SAR) on ALOS and ALOS-2 can observe the land surface even under clouds, and therefore the L-band SAR data have been providing useful information about forest changes in tropical region. The global 25m resolutions PALSAR/PALSAR-2 mosaic is a global SAR image created by mosaicking the SAR images in backscattering coefficients measured by PALSAR/PALSAR-2. Correction of geometric distortion specific to SAR (ortho-rectification) and topographic effects on image intensity (slope correction) are applied to make forest classification easy. The size of one pixel is approximately 25 meters by 25 meters. The temporal interval of the mosaic is generally 1 year. Global 25m resolution JERS-1 (Japanese Earth Resources Satellite-1) SAR mosaic dataset has been added since October 31, 2016. The original data of the mosaic were mainly acquired in 1996. The mosaic was generated with the same method as the PALSAR-2/PALSAR mosaic.
