# Supplementary material for: Association between residential greenspace structures and frailty in a cohort of older Chinese adults
Source: Commun Med (Lond). 2022 Apr 20;2:43. doi: 10.1038/s43856-022-00093-9 (PMC9053290; doi:10.1038/s43856-022-00093-9)
Supplement: Supplementary file 7 — Description of Additional Supplementary Files [file 43856_2022_93_MOESM7_ESM.pdf]

Description of Additional Supplementary Files

File Name: Supplementary Data 1

Description: Baseline Characteristics of CLHLS participants (N = 8,776).

NDVI: normalized difference vegetation index. LPI: largest patch index. Shape: shape index. Cohesion: patch cohesion index. N: numbers. FI: frailty index. P: p-value of One Way ANOVA of mean value differences between groups.

File Name: Supplementary Data 2

Description: Serial cross-sectional analysis (N = 8,776) and longitudinal analysis (N = 2,855) of the association between residential greenness structures and frailty.

Adjusted for age, sex, the study entrant year, marital status, geographic region, urban or rural residential location, literacy, annual household income, financial independence, BMI, smoking status, alcohol consumption, exercise status, PM2.5. NDVI: normalized difference vegetation index. LPI: largest patch index. Shape: shape index. Cohesion: patch cohesion index. N: numbers. Coef: coefficient. CI: confidence interval. OR: odds ratio. Q1~Q4: quartiles. *p<0.05. **p<0.01. *** p<0.0001.

File Name: Supplementary Data 3

Description: Sensitivity analysis of frailty and other indices of greenspace structures in China.

Age, sex, the study entrant year, marital status, geographic region, urban or rural residential location, literacy, annual household income, financial independence, BMI, smoking status, alcohol consumption, exercise status, PM2.5. ED: edge density. FRAC: fractal dimension index. PLADJ: percentage of like adjacencies. N: numbers. Coef: coefficient. CI: confidence interval. OR: odds ratio. *p<0.05. **p<0.01. *** p<0.0001.

File Name: Supplementary Data 4

Description: Source data of Figure 2
